# Hematological- and Neurological-Expressed Sequence 1 Gene Products in Progenitor Cells during Newt Retinal Development

**DOI:** 10.1155/2012/436042

**Published:** 2012-06-06

**Authors:** Tatsushi Goto, Fumio Tokunaga, Osamu Hisatomi

**Affiliations:** Department of Earth and Space Science, Graduate School of Science, Osaka University, Machikaneyama-cho 1-1, Toyonaka 560-0043, Japan

## Abstract

Urodele amphibians such as Japanese common newts have a remarkable ability to regenerate their injured neural retina, even as adults. We found that hematological- and neurological-expressed sequence 1 (Hn1) gene was induced in depigmented retinal pigment epithelial (RPE) cells, and its expression was maintained at later stages of newt retinal regeneration. In this study, we investigated the distribution of the HN1 protein, the product of the Hn1 gene, in the developing retinas. Our immunohistochemical analyses suggested that the HN1 protein was highly expressed in an immature retina, and the subcellular localization changed during this retinogenesis as observed in newt retinal regeneration. We also found that the expression of Hn1 gene was not induced in mouse after retinal removal. Our results showed that Hn1 gene can be useful for detection of undifferentiated and dedifferentiated cells during both newt retinal development and regeneration.

## 1. Introduction

The vertebrate retina is a tissue specialized in receiving photons and in generating electrical signals which are sent to the brain. The neural retina is a highly organized neural tissue, which is unable to be regenerated in mammals. In contrast, Urodele amphibians such as Japanese common newts (*Cynops pyrrhogaster*) have an amazing ability to regenerate their retina, even as adults [1, 2]. Studies on this process of retinal regeneration may help to understand the mechanism of neural retina formation and to rescue patients from blindness.

The regeneration process of newt retinas has been investigated morphologically for many years [3, 4]. When the neural retinas are surgically removed from newt eyes, the regeneration takes place in the following order [5, 6]. First, the remaining retinal pigment epithelial (RPE) cells lose their pigment granules (depigmentation) and transdifferentiate into retinal progenitor cells [7–9]. Subsequently, these retinal progenitor cells proliferate and differentiate into various neurons and glial cells to reconstruct a neural retina [10]. The retinal regeneration processes usually complete within 5-6 weeks, and newts recover their vision at about 3 months when the optic nerve connects to visual centers in the brain [11]. Although it has been suggested that most processes of retinal regeneration are similar to that of retinal development [10, 12–15], the dedifferentiation of RPE cells is a unique and important event for regeneration. 

We carried out differential display analyses and found that the hematological, and neurological-expressed sequence 1 (Hn1) gene was upregulated within a few days after removal of the retina [16]. The Hn1 gene was first identified by Tang and colleagues in 9-day-old mouse embryos and was reported to be expressed more abundantly in the brain and hematopoietic cells of embryos than those of adults [17]. Genes homologous to Hn1 have been found in genomes of not only vertebrates, but also invertebrates, forming an evolutionarily conserved gene family [18]. It has been reported that the products of *jupiter*, a *Drosophila* homolog of vertebrate Hn1, bound to the microtubule both *in vitro* and *in vivo* [19]. In vertebrates, Zujovic and the coworkers identified that the rodent Hn1 genes were associated with spontaneous regeneration of motoneurons [20]. Laughlin and the coworkers performed the knock-down analysis of Hn1 gene using melanoma cell line B16.F10, suggesting that the Hn1 gene acted as a suppressor of differentiation in proliferating cells [21]. In the present study, we investigated the expression of the Hn1 gene during retinal formation and discussed the role of its translated products (HN1 protein).

## 2. Materials and Methods

### 2.1. Animals

Adult Japanese common newts (*Cynops pyrrhogaster*) were obtained from Hamamatsu Seibutsu Kyozai (Hamamatsu, Japan). Newt larvae were bred as previously described [22]. Mice, the strain name of C57BL/6J, were purchased from Nippon Doubutsu Co. Ltd., (Osaka, Japan). Under general anesthesia using Nembutal (Dainippon Sumitomo Pharma Co. Ltd., Osaka, Japan), eyes were incised along dorsal half of corneal limbus. The incision was sutured after the removal of lens and retina. The mice were kept in normal cages till euthanasia by carbon dioxide gas. The newts and mice were handled according to the Guidelines for Animal Experiment of Osaka University.

### 2.2. Immunohistochemistry

Whole newt embryos were fixed as previously described [22]. The developmental stages of embryos were determined according to the criteria by Okada and Ichikawa [23]. Cryosections of 8–10 *μ*m thickness were dried at 65°C and fixed again with 4% paraformaldehyde in PBS. Sections were blocked with block solution (PBS containing 3% BSA, 1% goat serum, and 0.4% Triton X-100), and incubated with anti-HN1 antiserum diluted 1 : 100 with block solution at 4°C overnight. After washing with PBST (0.1% Tween20 in PBS) several times, sections were incubated with Alexa Fluor488-conjugated anti-mouse IgG (life technologies) diluted 1 : 500 with PBST, for 1 h at room temperature. The antiserum preincubated with recombinant HN1 at 4°C overnight was used as negative control. For staining of cell nuclei, sections were incubated with 1 *μ*g/mL ethidium bromide for 15 min in PBST. Fluorescent signals were detected using the fluorescent microscope BX50 (Olympus, Tokyo, Japan) or the confocal microscope system FLUOVIEW (Olympus).

### 2.3. Overexpression of HN1 in COS7 Cells

The complete coding region of Hn1 cDNA was inserted between EcoRI and XhoI sites of a mammalian expression vector, pcDNA3 (Life Technologies). COS7 cells were diluted to 1–5 × 10^4^ cells/dish on a *ϕ*60 mm dish at 37°C in a 5% CO_2_ incubator. For immunocytochemical analyses, coverslips (*ϕ*30 mm) were placed on the bottom of the dishes before the dilution. Next, the constructs were introduced with FuGENE6 (Roche Diagnostics K. K.) according to manufacturer's protocol. The empty pcDNA3 vector was transfected as a negative control. 

For immunohistochemistry of COS7 cells, samples were fixed with 4% paraformaldehyde in PBS at 48 h after the transfection. We performed the whole-mount immunohistochemical staining by the method described above for cryosections, with the following alterations : (1) cells were incubated with anti-HN1 antiserum diluted 1 : 100 and with antitubulin *α* antibodies (Lab Vision Co., Fremont, CA) diluted 1 : 150 with block solution; (2) biotinylated anti-rabbit IgG (1.5 mg/mL, Vector Laboratories Inc., Burlingame, CA, USA) diluted 1 : 100 was used as a secondary antibody, and the cells were then washed gently with PBST and treated with Cy 3-conjugated streptavidin (Jackson ImmunoResearch Laboratories, Inc., West Grove, PA, USA) solution for the detection of tubulin; (3) the cells were treated with 0.5 *μ*g/mL of Hoechst33258 (Life Technologies) for nuclear staining.

### 2.4. RT-PCR Analysis

Total RNAs were eluted by ISOGEN (Nippongene Co. Ltd., Tokyo, Japan) from mice eyecups after retinal removal. Subsequently, cDNAs were synthesized using Reverse Transcriptase M-MuLV (Roche Diagnostics K. K., Tokyo, Japan) and a pd(T)_12–18_ (GE Healthcare), and used as template for PCR amplification of Hn1 and *β*-actin (internal control). Oligonucleotide primers were designed as follows: mHn1rtf1, 5′-GCCTCGAGGTGGGTCCAATTTTTCATT-3′, mHn1rtr1, 5′-GCGTCGACTGGCTTCTCCTCCATCTG-3′, for amplification of mouse Hn1 cDNA; and mActinf1, 5′-GAACATGGCATTGTTACCAACTGG-3′, mActinr1: 5′-AGCATAGCCCTCGTAGATGGGC-3′. Thirty-five cycles of PCR were carried out at 94°C for 30 sec, 56°C for 30 sec, and 72°C for 60 sec.

## 3. Results

### 3.1. Expression of the HN1 Protein in Developing Newt Retinas

**Figure 1:**
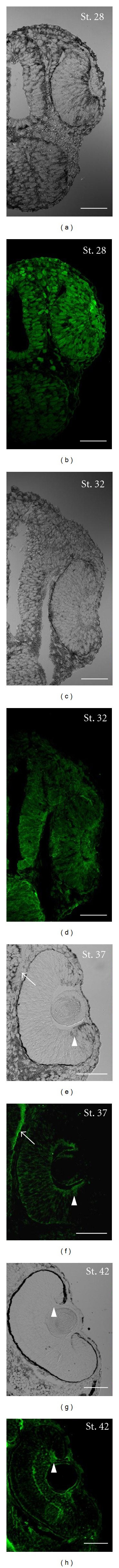
Immunoreactivities for HN1 protein in newt retinas at early stages of eye development. The developmental stages of embryos were determined as shown in, Materials and Methods. Nomarski images (a, c, e, and g) and immunoreactivity (b, d, f, and h) in transverse sections of stage 28 (a, and b) and stage 32 (c, and d) embryos, and in coronal sections of stage 37 (e, and f) and stage 42 (g, and h) embryos. The fibrillary fluorescent signal was observed, corresponding to the region outside RPE layer from the bright-field observation (arrows in Figures 1(e) and 1(f)). All scale bars indicate 100 *μ*m.

Our previous study showed that the Hn1 gene was induced in the RPE cells at an early stage during newt retinal regeneration [16]. To clarify the distribution of HN1 protein during retinal formation, we performed immunohistochemical analyses for the HN1 protein in newt embryos. At developmental stage 28, optic cups evaginated from a neural tube. The immunoreactivity of our anti-HN1 antiserum was observed not only in whole eyes, but also in presumptive brain and gut (Figures 1(a) and 1(b)). At developmental stage 32, the eyes grew larger and the epidermis contacting the retinas invaginated to form lenses. The immunoreactivity for the HN1 protein was still observed in the whole retinas (Figures 1(c) and 1(d)). At developmental stage 37, when a lens and a neural retina consisting of multiple-layered cells were formed, the anti-HN1 antiserum recognized the marginal region of each cell (Figures 1(e) and 1(f)). At developmental stage 42, when plexiform layers and a RPE layer were formed, the immunoreactivity for the HN1 protein was observed particularly in the plexiform layers (Figures 1(g) and 1(h)). The HN1 immunoreactivities were also found at the medial side in the lateral region (arrowheads in Figures 1(e)–1(h)). The HN1-immunopositive cells may be retinal progenitor cells in ciliarly marginal zone. These data indicated that the HN1 protein was highly expressed in undifferentiated cells during normal retina development.

**Figure 2:**
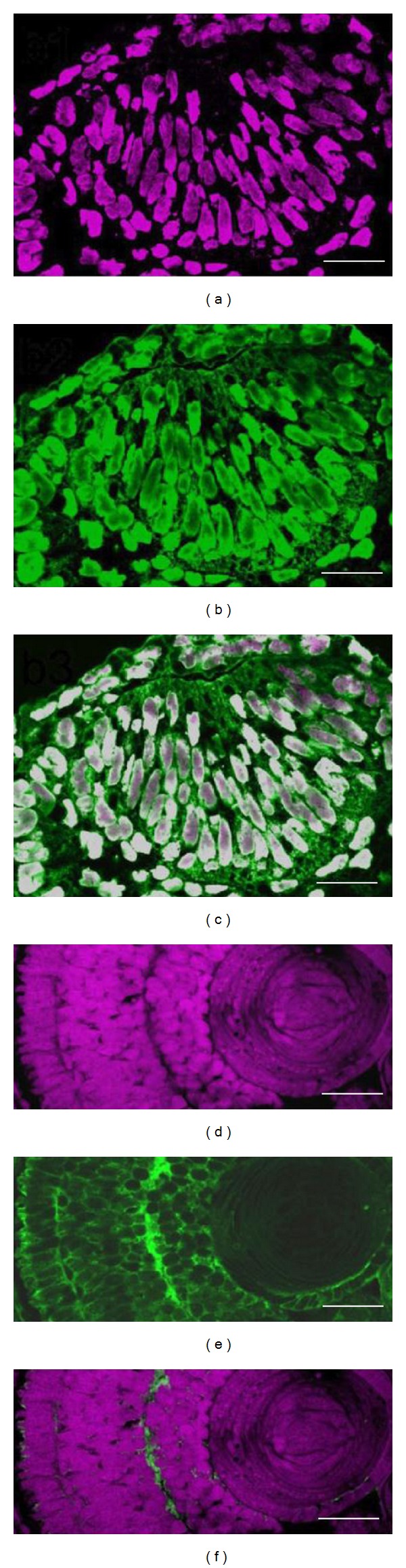
Double staining experiment of a developing retina at stage 28 (a–c) and stage 42 (d–f). Cell nuclei (a and d), the immunoreactivity for the HN1 protein (b and e), and the composite image (c and f). All scale bars indicate 50 *μ*m.

Then, we examined detail of the HN1 protein in the developing newt retina by double-staining experiments (Figure 2). The immunoreactivity for the HN1 protein was overlapped with the cell nuclei in immature retinas at the developmental stage 28 (Figures 2(a)–2(c)). While the immunoreactivity for the HN1 protein was not observed in the cell nuclei at the later developmental stage 42 (Figures 2(d)–2(f)). These results were consistent with our previous study on the regenerating newt retina [16], indicating that the HN1 protein plays similar roles during development and regeneration of newt neural retinas.

### 3.2. Overexpression of Newt HN1 Protein in Cultured Cells

**Figure 3:**
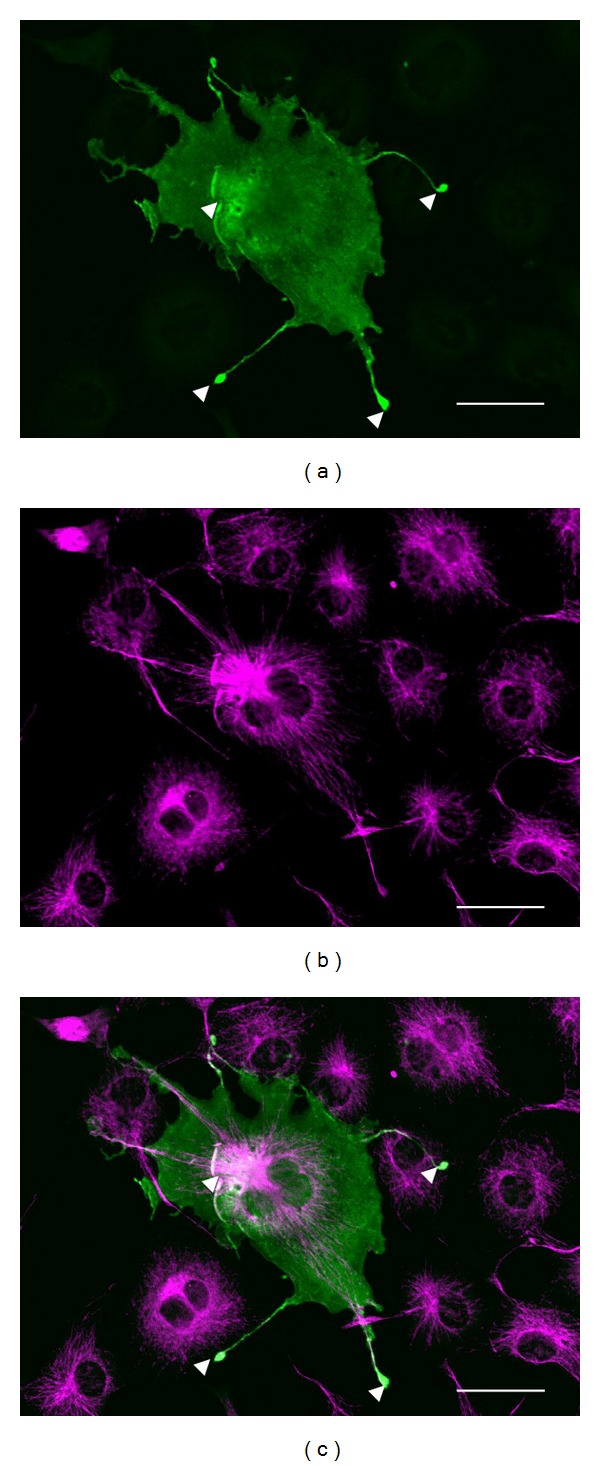
Subcellular localization of newt HN1 protein in COS7 cells. (a) shows the immunoreactivity for the HN1 protein. (b) shows the immunoreactivity for tubulin. (c) shows the composite image of (a) and (b). Arrowheads indicate the relatively high immunoreactive signal of HN1. All scale bars indicate 50 *μ*m.

Overexpression analysis of the uncharacterized protein in cultured cells can be a useful approach to solve the protein function. We introduced the Hn1 gene into COS7 cells and investigated the immunoreactivity of our anti-HN1 antiserum. It was indicated that these signals are derived from the exogenous newt HN1 protein, not from the intrinsic HN1 protein, since no signals were observed in negative control experiment. The immunoreactivities were entirely observed in the COS7 cells (Figure 3(a)). Especially, the relatively high immunoreactivities were observed in the cytosol and cell processes (arrowheads in Figure 3(a)). Karpova and the coworkers showed that the products of *Jupiter* gene had binding ability to microtubule [19]. Hence, we examined whether the overexpression of newt HN1 protein affected to the cytoskeleton of COS7 cells. The fiber-like immunoreactive signals for tubulin were observed, which showed a different pattern from that for the HN1 protein (Figure 3(b)). The microtubule pattern was undistinguishable from the surrounding cells that lacked the exogenous HN1 protein (Figure 3(c)), suggesting that the overexpression of the HN1 protein did not affect to the microtubule network.

### 3.3. Expression of Mouse Hn1 mRNA in RPE Cells after Retinal Removal

**Figure 4:**
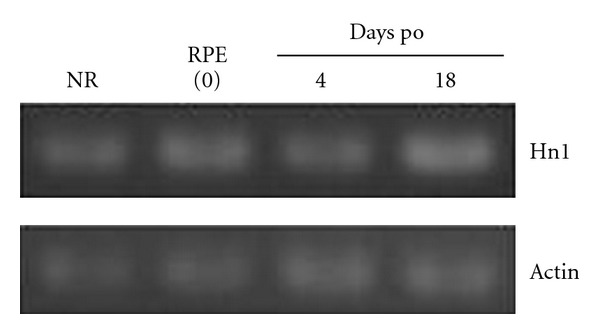
RT-PCR analysis of mouse Hn1 gene after retinal removal. The upper panel shows the expression of mouse Hn1 gene, and the lower panel shows the expression of the *β*-actin gene as an internal control. Abbreviations indicated as follows: NR: normal retina from adult mice; RPE: retinal pigment epithelium from adult mice; days po: days after operation of retinal removal.

We used mice as a model which lacked the ability of spontaneous regeneration. Four days after the removal of neural retinas from mouse eyes, most pigmented cell layers were attached to sclera, but some part of cell layers was detached, and pigmented cells were found inside of eye cavity (data not shown). The appearance of pigmented cell layers was similar at 18 days after operation. Our observation did not contradict the previous report that the retinectomy caused the migration of the epithelial cells, development of multiple layers with vacuoles in rabbit eyes [24]. We examined the expressions of Hn1 gene in mouse eyecups by RT-PCR (Figure 4). Hn1 mRNA was expressed in mouse normal retina and RPE cells. After retinal removal, the expression level of mouse Hn1 gene was almost equal, suggesting that mouse Hn1 gene did not induced the remaining RPE cells or others, unlike in the case of newt. The upregulation of Hn1 gene was possibly a unique phenomenon observed in dedifferentiating RPE cells.

## 4. Discussion

The important finding in our studies was that the subcellular localization of the newt HN1 protein changed in accordance with maturation of newt retinas both in retinal development (Figure 2) and regeneration [16]. Zhou and colleagues performed an overexpression analysis of a human HN1 protein fused with GFP in HeLa cells. They have suggested that the GFP signal was accumulated in the nuclei and therefore proposed that the HN1 is a nucleoprotein [18]. They predicted the consensus sequence (PVRKNKM) for nuclear localization of the HN1 protein by sequence analysis. The newt HN1 protein has the same sequence (PVRKHKM) as human HN1 protein at a corresponding position, and the immunoreactivity for the HN1 protein was overlapped with the cell nuclei at the developmental stage 28 (Figure 2). 

In contrast, fluorescent microscopic observation in the transgenic fruit fly that expressed Jupiter-GFP fusion protein showed that the Jupiter localized in microtubule network through the cell cycle [19]. Isolated recombinant Jupiter-GFP proteins were indicated to bind directly to microtubule or to microtubule-associated proteins. The immunoreactivity for the newt HN1 protein was not localized in cell nuclei at the later developmental stages (Figure 2) and in COS7 cells (Figure 3). It should be noted that the relatively high immunoreactivities were observed at the tips of processes and along with the microtubules in COS7 cells (Figure 3(c), arrowheads). It seems possible that the expression level of HN1 protein in COS7 cells was high enough to saturate the binding site of tubulin, and the signal from unbound HN1 was observed both in the cytosol and the nucleus without affecting the appearance of cytoskeleton. HN1 protein may play multiple roles during formation and maintenance of neural tissues.

Laughlin and coworkers performed an interesting study about function of vertebrate Hn1 gene [21]. They investigated the effect of gene suppression of intrinsic Hn1 in melanoma B16.F10 cells. B16.F10 cells have a melanogenesis activity like RPE cells. Introduction of specific siRNA against Hn1 gene caused melanogenesis in B16.F10 cells with an increase of expression about melanogenic protein, tyrosinase and TRP2, and with a promotion of the interaction between actin and Rab27a proteins which were involved in the secretion pathway of melanosoma. They also showed that the knockdown of Hn1 induced a G1/S cell-cycle arrest from western blot analyses about the factors involved in a cell-cycle and a cell-growth signaling. Therefore, they concluded that the Hn1 gene inhibited the cell differentiation. It was consistent with Laughlin's results that the strong immunoreactivities for newt HN1 protein were observed in immature retinas showing active proliferation of retinal progenitor cells (Figure 1). 

The Hn1 family genes were found in the genomes of various vertebrates and invertebrates. However, the function of Hn1 was uncharacterized in most animals. The expression of rodent Hn1 was compared by Zujovic and coworkers among four different axotomy models: the facial nerve axotomy in the adult and the newborn, vagotomy, and rubrospinal tractotomy, which showed that rodent Hn1 was upregulated only in some peripheral neurons which can regenerate spontaneously [20]. Previously, we showed that the newt Hn1 gene was upregulated after retinal removal by RT-PCR [16]. However, our RT-PCR analysis in this study showed that mouse Hn1 gene was not induced after retinal removal (Figure 4). These results could support a hypothesis that the expression of animal Hn1 gene correlated the ability for neural regeneration in vertebrates. 

Our immunohistochemical analyses showed that the HN1 protein strongly expressed in pluripotent cells in normal development. The expression of the newt Hn1 gene is upregulated in depigmented RPE cells at early stages of retinal regeneration [16], implying that the depigmented RPE cells has a character like stem cells. The upregulation of the Hn1 gene is preceded to those of transcription factors, Ngn-1, and Pax-6, important for retinal formation [6, 25]. The Hn1 gene may be an available maker to detect dedifferented cells or stem cells in adult tissues.

## 5. Conclusion

The expression of the newt HN1 protein is highly activated at early stages of retinal formation during development and regeneration and is not upregulated in mouse RPE cells lacking the ability to be dedifferentiated into the progenitor cells. It was also suggested that the HN1 protein changes its subcellular localization from nuclei to cytosol during formation of newt neural retina. The upregulation of the Hn1 gene may be an available maker to detect dedifferented cells or stem cells in adult tissues.
